# Herpetofauna of the Northwest Amazon forest in the state of Maranhão, Brazil, with remarks on the Gurupi Biological Reserve

**DOI:** 10.3897/zookeys.643.8215

**Published:** 2017-01-06

**Authors:** Marco Antonio de Freitas, Ruhan Saldanha Vieira, Omar Machado Entiauspe-Neto, Samantha Oliveira e Sousa, Tayse Farias, Alanna Grazieli Sousa, Geraldo Jorge Barbosa de Moura

**Affiliations:** 1 Instituto Chico Mendes de Conservação da Biodiversidade (ICMBio), PARNA Catimbau, Vila Catimbau, CEP 56537-000, Buíque, PE, Brazil; 2 Instituto Chico Mendes de Conservação da Biodiversidade, (ICMBio). REBIO Gurupi, BR 222-KM 12. CEP 65930-000. Pequiá, Açailândia, MA, Brasil; 3 Universidade Federal do Rio Grande, Instituto de Ciências Biológicas, Laboratório de Vertebrados, Av. Itália Km 8, CEP: 96203-900, Vila Carreiros, Rio Grande, Rio Grande do Sul, Brazil; 4 Universidade de Ensino Superior do Sul do Maranhão (UNISULMA), Departamento de Ciências Biológicas, Rua São Pedro, 11, Jardim Cristo Rei, CEP 65907-070, Imperatriz, MA, Brazil; 5 Centro Universitário do Estado do Pará (CESUPA), Unidade José Malcher, Departamento de Biologia, Avenida Governador José Malcher, 1963, CEP 66060-230, Belém, PA, Brazil; 6 Universidade Federal Rural de Pernambuco (UFRPE), Departamento de Biologia, Laboratório de Estudos Herpetológicos e Paleoherpetológicos – LEHP, Rua Don Manuel de Medreiros S/N, Dois Irmãos, CEP 81840-000, Recife, PE, Brazil. Programa de Pós-graduação em Ecologia-UFRPE. Programa de Pós-graduação em Ciência Animal Tropical-UFRPE

**Keywords:** Amphibians, Belém Center of Endemism, inventory, new records, reptiles

## Abstract

Understanding the biodiversity of an area is the first step for establishing effective interventions for conservation, especially when it comes to herpetofauna, since 4.1% and 9.2%, respectively, of Brazilian amphibians and reptiles are endangered. The aim of this study is to identify the composition of the herpetofauna occurring in the Northwest Amazonian state of Maranhão, with a focus on the Gurupi Biological Reserve and surrounding areas. Samples were collected between May 2012 and October 2013 (18 months), through pitfall traps, time constrained active search, and opportunistic encounters, and these records were supplemented by specimens collected by third parties and by bibliographic records. A total of 131 species were recorded: 31 species of amphibians and 100 species of reptiles (six testudines, 30 lizards, two amphisbaenas, 60 snakes and two alligators), including some species new to the state of Maranhão and the northeast region of Brazil. This inventory contributes to the knowledge of the herpetofauna for the Belém Endemism Center, the most devastated region of the Brazilian Amazon, and considered poorly sampled.

## Introduction

Conservation units in the Amazon (National Parks, ecological stations, extractive reserves, national forests, biological reserves, etc.) are of fundamental importance for the conservation of biodiversity in this biome (Peres 2005; Silva 2005).

With regard to formulating effective management plans, it is of fundamental importance for biodiversity conservation to know the composition of the fauna and flora. Faunal lists, especially in regions that represent sampling gaps and that refer to groups with a high percentage of endangered species should be emphasized (Peres 2005).

In this context, regarding the herpetofauna, there are in Brazil 1080 known species of amphibians and 773 species of reptiles of which 4.1% and 9.2% are endangered, respectively (Costa and Bérnils 2015; Segalla et al. 2016).

Biodiversity inventories enable us to identify the degree of regional endemism, new trigger points, new species and the actual conservation status of the species recorded, besides enriching regional and national scientific collections (Moura et al. 2014).

It is noteworthy that species lists are highly important for our understanding of the environmental conservation, since the occurrence of bioindicator species (either opportunistic or vulnerable) can be used to determine the effects of environmental disturbance in the area, and therefore, pave a way to efficient interventions and conservation policies (Brown and Freitas 2002).

The state of Maranhão is known for its rich biodiversity (Martins and Oliveira 2011), lack of researchers and extensive sampling gaps, especially regarding the herpetofauna in the Amazon biome of northwestern Maranhão.

Among the indicators of the need for wildlife studies in Maranhão are the articles of the last decades that describe new species in Amazonia, especially in the eastern Amazon (Caramaschi 2010; Vaz-Silva et al. 2015), as well as the few publications, concentrated mainly on snakes (Cunha and Nascimento 1993; Freitas et al. 2014), lizards (Avila-Pires and Vitt 1998; Avila-Pires 1995; Freitas et al. 2013) and anurans (Andrade et al. 2003; Rodrigues et al. 2003; Andrade et al. 2011; Barreto et al. 2011; Miranda et al. 2012; Miranda et al. 2013; Matavelli et al. 2013; Freitas et al. 2014b, c, d; Vaz-Silva et al. 2015).

This study aims to record the composition of amphibians and reptiles of the REBIO Gurupi and adjacent regions of the northwest Amazon of Maranhão state, a conservation unit of utmost importance, composing the largest and last block of continuous forests in the Belém Endemism Center (Martins and Oliveira 2011).

## Materials and methods

**Figure 1.**
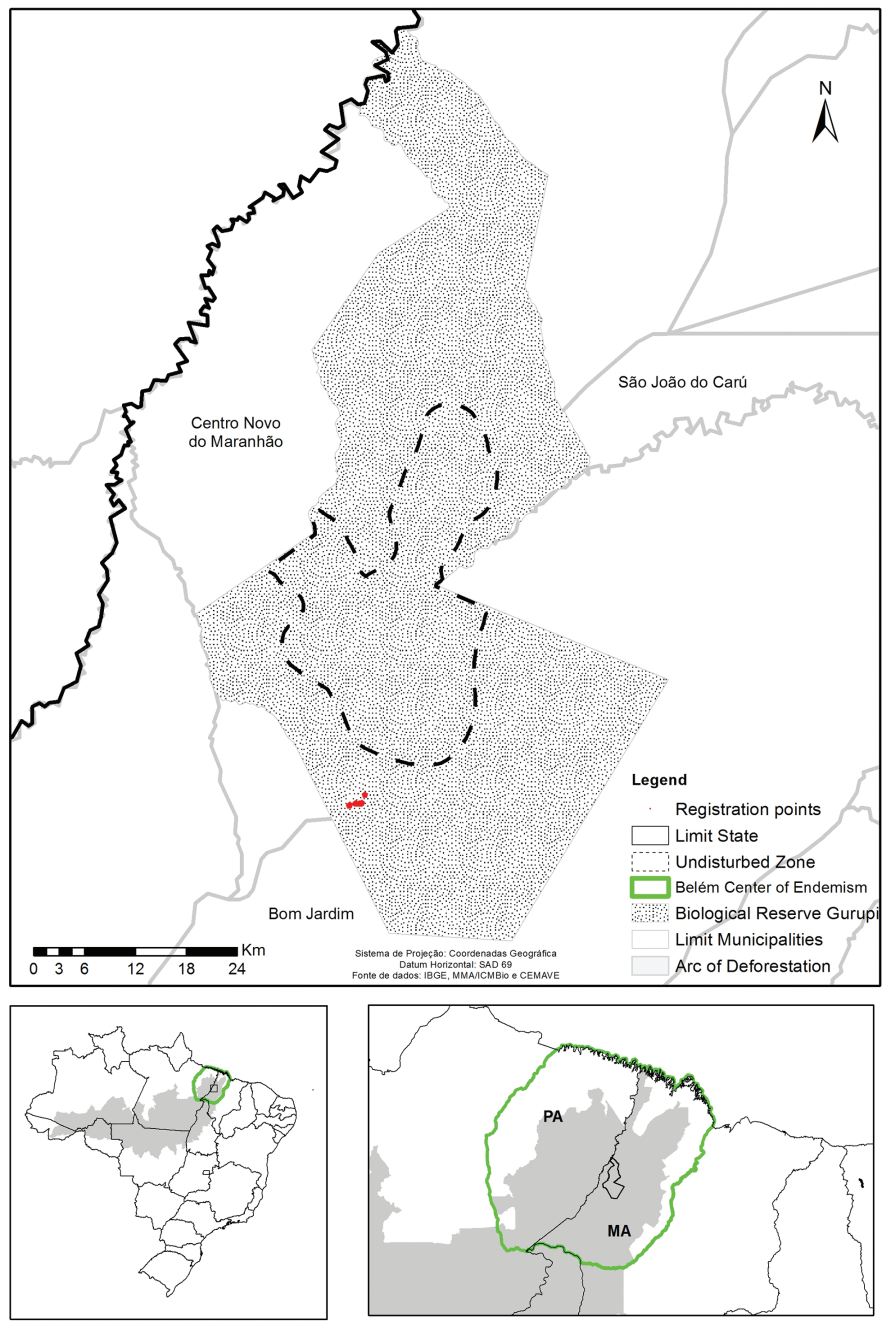
Map with the location of the study area, in the Gurupi Biological Reserve, Maranhão, Brazil.

Study Area: The Gurupi Biological Reserve (03°58'32"S 46°46'52"W) (Figure 1), was created by Law 95 614 1988 and occupies an original area of 341,650 hectares in the municipalities of Bom Jardim, Centro Novo do Maranhão and São João do Caru, in the state of Maranhão (IBAMA 2006). The climate is humid type B2, with moderate water deficit in the summer, average annual rainfall exceeding 2,000 and average temperature greater than 24°C, with the soil type being argisoil (Martins and Oliveira 2011). This work was performed and focused only in Gurupi and the adjacent rainforests of the State of Maranhão, northwest Amazon.

Sampling: Samples were collected between May 2012 and October 2013 (18 months), with pitfall traps, time constrained active search, and opportunistic encounters in the forested areas and access roads to REBIO (road killed individuals). They also included those collected by third parties and bibliographic records.

The pitfall traps were composed of seven lines of five buckets (60 L) connected by a plastic canvas with a length of 10m, totaling 70 meters of intercept lines (Line 1: 03°59'14"S, 46°47'53"W; Line 2: 03°59'11"S, 46°47'50"W; Line 3: 03°59'05 “S, 46°47'27"W; Line 4: 03°59'06"S, 46°47'25 “W; Line 5: 03°59'07"S, 46° 47'15"W; Line 6: 03°59'03"S, 46°47'04"W; Line 7: 03°58'32"S, 46°46'52"W), each line was about 3 km distant from one another. Traps were open around the clock for five days during each sampling campaign. These were concentrated in the rainy season, December 2012, January and May 2013, totaling an effort of 5400 hours/ bucket.

The time-constrained active search was conducted over three campaigns for three nights in December 2012, January and May 2013, by a team made up of five collectors, totaling an effort of 135 man hours according to the methodologies of Martins and Oliveira (1998), and Bernarde and Abe (2006). The search was concentrated in the area adjacent to aquatic environments near the southern tip of REBIO Gurupi (04°00'20"S, 46°46'41"W).

The collected amphibians were euthanized by applying lidocaine ointment (lidocaine) on the ventral region and the reptiles through overdosing with ether (Callefo 2002; Franco and Salomão 2002). All specimens were fixed with 10% formalin and then preserved in 70% ethanol.

Voucher specimens were deposited in the collection of Herpetology and Paleontology of the Federal Rural University of Pernambuco CHP-UFRPE (tumble numbers in Appendix 1) and the University of São Paulo Museum of Zoology MZUSP (tumble numbers in Appendix 1). Important bibliographic databases added to this work were Cunha and Nascimento (1993) for snakes, Avila-Pires (1995) for lizards and Barreto et al. (2011) for amphibians and reptiles.

Data analysis: In order to evaluate the efficiency of the collection effort in the analyzed fragment, species rarefaction curves were constructed with 1000 randomizations generated based on the data matrix of the relationship between richness and abundance (ICE index) through the statistical program Ecosim version 7.0 (Gotelli and Enstminger 2003).

## Results

**Figure 3.**
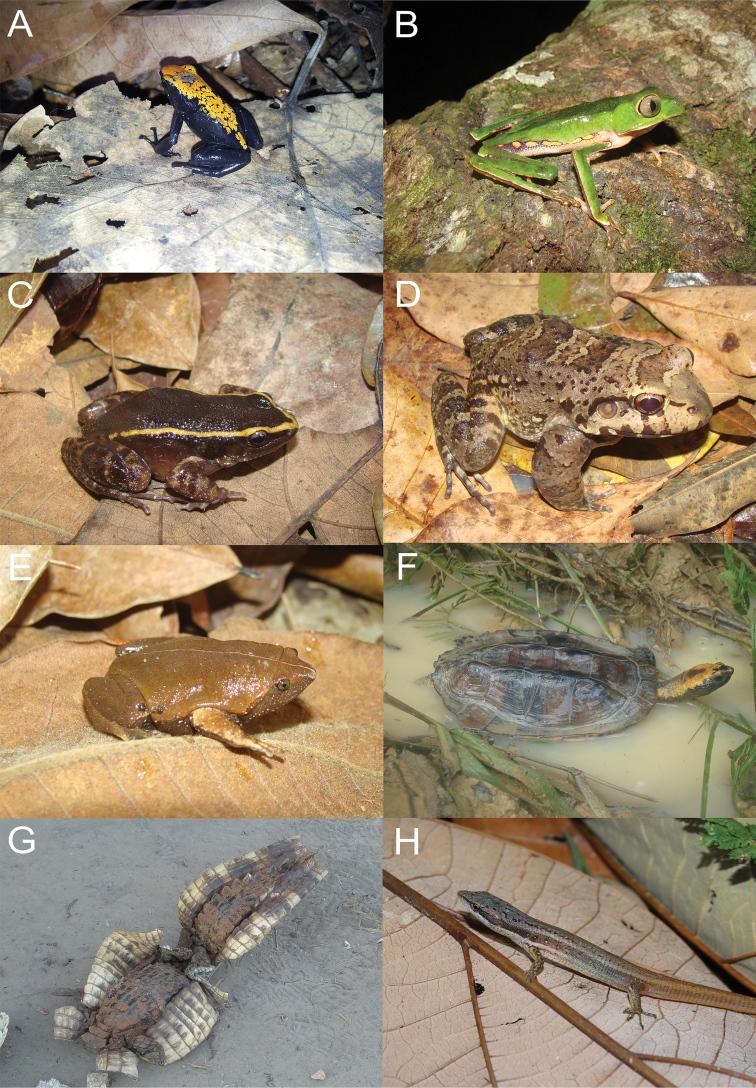
Some amphibians and reptiles recorded in the Gurupi Biological Reserve, Maranhão, Brazil. **A** *Adelphobates galactonotus* **B** *Phyllomedusa vaillanti* **C** *Lithodytes lineatus* **D** *Leptodactylus paraensis* **E** *Ctenophryne geayi* **F** *Platemys platicephala* **G** *Paleosuchus trigonatus* **H** *Cercosaura argulus*. Photo credit of *Paleosuchus trigonatus* (**G**) belong to Eloisa Mendonça and M. A. de Freitas for others.

**Figure 4.**
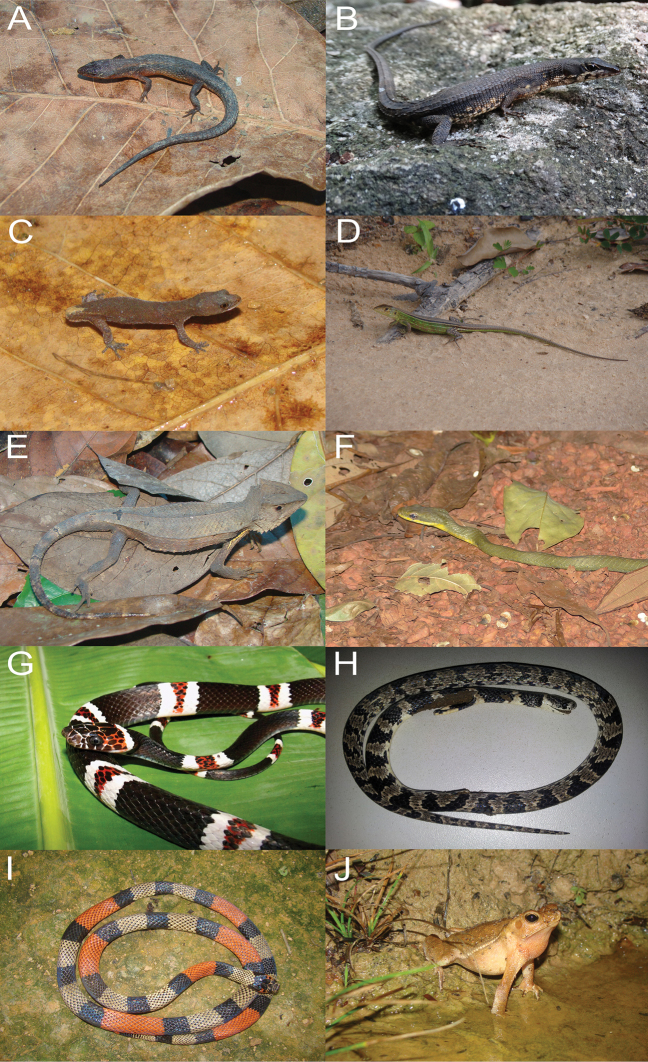
Some amphibians and reptiles recorded in the Gurupi Biological Reserve, Maranhão, Brazil. **A** *Leposoma percarinatum* **B** *Neusticurus bicarinatus* **C** *Coleodactylus septentrionalis* **D** *Cnemidophorus cryptus* **E** *Stenocercus dumerilii* **F** *Phrynonax poecilonotus* **G** *Rhinobothryum lentiginosum* **H** *Sibynomorphus mikanii septentrionalis* **I** *Micrurus spixii martiusi* **J** *Rhinella gildae*. Photo credits of *Phrynonax poecilonotus* (**F**) belong to Eloisa Mendonça, *Rhinobothryum lentiginosum* (**G**) to Saymon Albuquerque and M. A. de Freitas for others.

A total of 131 species composing the local herpetofauna was recorded: 31 species of amphibians (seven families) and 100 species of reptiles, which included six testudines, 30 lizards, two amphisbaenians, 60 snakes and two crocodylians (Figure 3, 4).

Of the 131 species recorded for the northwest Amazon in Maranhão, 78 species (62%) were recorded in this study and 53 (38%) included results from previously published data (Cunha and Nascimento 1993; Avila-Pires 1995; Barreto et al. 2011).

## Discussion

The Belém Endemism Center (EC) is the smallest among the eight EC in the Amazon region, and has the largest loss of forest cover, with about 70% of its total area already destroyed (Silva et al. 2005; Fearnside 2006).

The diversity of amphibians and reptiles of the Belém Endemism Center is very high, as in other endemic centers, especially those of the eastern Amazon region, such as the ECs Guyana, Xingu and Tapajós: 85 species of snakes further down the Amazon River, in Santarem, state of Pará at EC Tapajós (Frota et al. 2005); 62 species of amphibians and reptiles in the Biological Reserve Tapirape, also at Pará, in the EC Xingu (Bernardo et al. 2012); 71 species of amphibians in the National Forest of Carajás, also at the EC Xingu (Pinheiro et al. 2012); 175 species of amphibians and reptiles in the National Forest of Saracá-Taquera, in theEC Guiana at Pará (Morato et al. 2014.); 53 species of snakes for the National Forest of Caxiuanã (at the EC Xingu (Santos-Costa et al. 2015); 78 species of amphibians and reptiles in the northern part of the state of Maranhão (Barreto et al. 2011). It is noteworthy that the last of these studies includes species of open areas, which are present due to the strong influence of Cerrado vegetation in northern Maranhão, as seen in the inventory of Miranda et al. (2012), which lists 42 species of reptiles for the coastal region of Maranhão, which is characterized by open vegetation and the predominance of coastal dunes.

The species richness of REBIO Gurupi is second only to the richness sampled in the National Forest of Saracá-Taquera in northern Pará, located in the Guyana Endemism Center, more than 1000 km from REBIO Gurupi (Morato et al. 2014).

**Figure 2.**
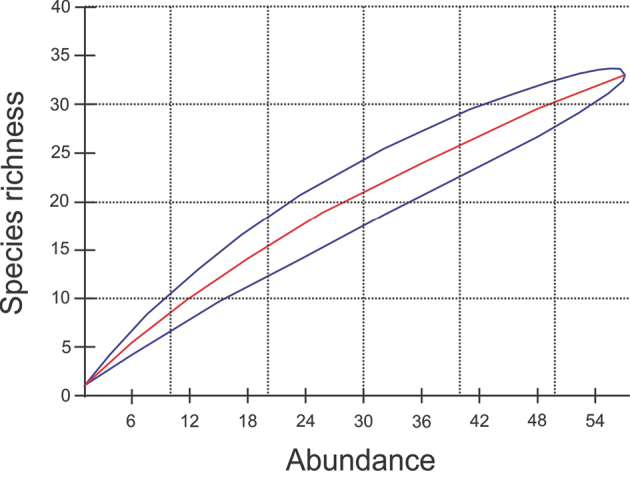
Thinning curves representing the accumulated richness of species of frogs and lizards at REBIO Gurupi. The richness was recorded by passive and active collection between December 2012 and May 2013. The center line is the average (randomizing 1000 times) and the lines on the side represent the associated standard deviation.

Regarding sampling efficiency, the collection effort and methods used were not effective, as not all niches were sampled. As a result, the curve implied 33 amphibian species and 78 reptile species at the site. As the curves did not reach an asymptote, we believe that site has potential for species that have not yet been recorded. This is borne out by the additional 58 species not found during the field work but recorded in the literature for the area (Cunha and Nascimento 1993; Avila-Pires 1995; Barreto et al. 2011) (Figure 2). Regarding collection methods, pitfall traps yielded only 33 species of the 78 found in the field, chiefly amphibians, frogs, and fossorial lizards.

## Conclusion

Since only 38% of the recorded species included in this inventory were obtained from literature (Table 1), it is possible to ascertain that our sampling reflects a great share of the total herpetofauna from Northwestern Maranhão. With this richness, the Gurupi Biological Reserve stands out as a biodiversity hotspot in the Amazon, reinforcing the need for its protection, and its position as the most important restricted use conservation unit of the Belém Endemism Center.

**Table 1.** Herpetofauna recorded between May 2012 and October 2013 in the Gurupi Biological Reserve, Maranhão, Brazil. (Type of record: EO = Opportunistic Encounter; BA = Constrained active search; Pitfalls = pitfall trap, DS = secondary data). * First occurrence for the state of Maranhão and northeast Brazil. ** Species endemic to the Belém center of endemism.

FAMILY	SPECIES	TYPE OF RECORD
Bufonidae	*Rhinella gildae* Vaz-Silva, Maciel, Bastos & Pombal Jr, 2015**	EO - DS (Vaz-Silva et al. 2015)
	*Rhinella marina* (Linnaeus, 1758)	EO/Pitfalls/BA
Craugastoridae	*Pristimantis fenestratus* (Steindachner, 1864)	BA
Dendrobatidae	*Adelphobates galactonotus* (Steindachner, 1864)**	BA
Hylidae	*Dendropsophus marmoratus* (Laurenti, 1768)	EO
	Dendropsophus cf. minusculus	DS (Barreto et al. 2011)
	*Dendropsophus nanus* (Boulenger, 1889)	DS (Barreto et al. 2011)
	*Dendropsophus minutus* (Peters, 1872)	DS (Barreto et al. 2011)
	*Dendropsophus rubicundulus* (Reinhardt & Lütken, 1862)	DS (Barreto et al. 2011)
	*Hypsiboas boans* (Linnaeus, 1758)	EO
	*Hypsiboas fasciatus* (Günther, 1859 “1858”)	DS (Barreto et al. 2011)
	*Hypsiboas punctatus* (Schneider, 1799)	BA
	*Hypsiboas multifasciatus* (Günther, 1859)	DS (Barreto et al. 2011)
	*Osteocephalus taurinus* Steindachner, 1862	BA
	*Scinax fuscomarginatus* (A. Lutz, 1925)	DS (Barreto et al. 2011)
	*Scinax nebulosus* (Spix, 1824)	DS (Barreto et al. 2011)
	*Scinax ruber* (Laurenti, 1768)	EO
	*Sphaenorhynchus lacteus* (Daudin, 1800)	DS (Benício et al. 2011)
	*Trachycephalus typhonius* (Linnaeus, 1758)	EO
	*Phyllomedusa hypochondrialis* (Daudin, 1800)	DS (Barreto et al. 2011)
	*Phyllomedusa vaillantii* Boulenger, 1882 *	BA (Freitas et al. 2014)
Leptodactylidae	*Adenomera andreae* (Müller, 1923)	BA/Pitfalls/EO
	*Leptodactylus fuscus* (Schneider, 1799)	EO
	*Leptodactylus mystaceus* (Spix, 1824)	BA/Pitfalls/EO
	*Leptodactylus rhodomystax* Boulenger, 1884	(Sá et al. 2014)
	*Leptodactylus paraensis* Heyer, 2005 ** -	EO
	*Leptodactylus troglodytes* A. Lutz, 1926	EO
	Leptodactylus gr latrans	DS (Barreto et al. 2011)
	*Lithodytes lineatus* (Schneider, 1799)*	(Freitas et al. 2014)
Leiuperidae	*Physalaemus cuvieri* Fitzinger, 1826	BA/Pitfalls
Microhylidae	*Ctenophryne geayi* Mocquard, 1904 *	(Freitas et al. 2014)
Chelidae	*Platemys platycephala* (Schneider, 1792) *	EO
Geoemydidae	*Rhinoclemmys punctularia* (Daudin, 1801)	EO
Kinosternidae	*Kinosternon scorpioides* (Linnaeus, 1766)	EO
Podocnemididae	*Podocnemis unifilis* Troschel, 1848	EO
Testudinidae	*Chelonoidis carbonarius* (Spix, 1824)	EO
	*Chelonoidis denticulatus* (Linnaeus, 1766)	EO
Alligatoridae	*Caiman crocodilus* (Linnaeus, 1758)	EO
	*Paleosuchus trigonatus* (Schneider, 1801) *	EO
Dactyloidae	*Dactyloa punctata* (Daudin, 1802)	BA
	*Norops fuscoauratus* (D’Orbigny, 1837)	BA/Pitfalls/EO
	*Norops tandai* (Ávila-Pires, 1995)	DS (Ávila-Pires 1995)
Gekkonidae	*Hemidactylus mabouia* (Moreau de Jonnès, 1818)	EO
Gymnophthalmidae	*Micrablepharus maximiliani* (Reinhardt & Luetken, 1862)	EO
	*Cercosaura argulus* Peters, 1863	(Freitas et al. 2013)
	*Cercosaura ocellata* Wagler, 1830	DS (Ávila-Pires 1995)
	*Colobosaura modesta* (Reinhardt & Luetken, 1862)	DS (Ávila-Pires 1995)
	*Arthrosaura reticulata* (O’Shaughnessy, 1881)	DS (Ávila-Pires 1995)
	*Leposoma percarinatum* Muller, 1923 *	Pitfalls
	*Neusticurus bicarinatus* (Linnaeus, 1758)**	DS (Ávila-Pires 1995)
Iguanidae	*Iguana iguana* (Linnaeus, 1758)	EO
Leiosauridae	*Enyalius leechii* (Boulenger, 1885)	DS (Ávila-Pires 1995)
Mabuyidae	*Copeoglossum nigropunctatum* (Spix, 1825)	Pitfalls
Phyllodactylidae	*Thecadactylus rapicauda* (Houttuyn, 1782)	EO
Polychrotidae	*Polychrus acutirostris* Spix, 1825	DS (Ávila-Pires 1995)
	*Polychrus marmoratus* (Linnaeus, 1758)	EO
Sphaerodactylidae	*Coleodactylus septentrionalis* (Vanzolini, 1980)**	Pitfalls
	*Gonatodes humeralis* (Guichenot, 1855)	Pitfalls/BA
Teiidae	*Ameiva ameiva* (Linnaeus, 1758)	Pitfalls/EO/BA
	*Cnemidophorus cryptus* Cole & Dessauer, 1993 *	EO
	*Kentropyx calcarata* Spix, 1825	Pitfalls/BA
	*Salvator merianae* (Duméril & Bibron, 1839)	EO/BA
	*Tupinambis teguixin* (Linnaeus, 1758)	DS (Ávila-Pires 1995)
	*Dracaena guianensis* Daudin, 1801	DS (Ávila-Pires 1995)
Tropiduridae	*Plica umbra* (Linnaeus, 1758)	Pitfalls
	*Stenocercus dumerilii* (Steindachner, 1867)**	Pitfalls
	*Tropidurus hispidus* (Spix, 1825)	EO/BA
	*Uracentron azureum* (Linnaeus, 1758)	DS (Ávila-Pires 1995)
	*Uranoscodon superciliosus* (Linnaeus, 1758)	BA
Amphisbaenidae	*Amphisbaena alba* Linnaeus, 1758	DS (Barreto et al. 2011)
	*Amphisbaena amazonica* Vanzolini, 1951	EO
Typhlopidae	*Amerotyphlops brongersmianus* (Vanzolini, 1976)	Pitfalls
Aniliidae	*Anilius scytale* (Linnaeus, 1758)	EO
Boidae	*Boa constrictor* Linnaeus, 1758	EO
	*Corallus hortulanus* (Linnaeus, 1758)	EO
	*Epicrates cenchria* (Linnaeus, 1758)	EO
	*Eunectes murinus* (Linnaeus, 1758)	DS (Cunha and Nascimento 1993)
Colubridae	*Chironius carinatus* (Linnaeus, 1758)	EO
	*Chironius exoletus* (Linnaeus, 1758)	EO
	*Chironius fuscus* (Linnaeus, 1758)	DS (Cunha and Nascimento 1993)
	*Chironius multiventris* Schmidt & Walker, 1943	DS (Cunha and Nascimento 1993)
	*Chironius scurrulus* (Wagler in Spix, 1824)	DS (Cunha and Nascimento 1993)
	*Drymarchon corais* (Boie, 1827)	EO
	*Drymoluber dichrous* (Peters, 1863)	DS (Cunha and Nascimento 1993)
	*Leptophis ahaetulla* (Linnaeus, 1758)	EO
	*Mastigodryas bifossatus* (Raddi, 1820)	DS (Cunha and Nascimento 1993)
Colubridae	*Mastigodryas boddaerti* (Sentzen, 1796)	EO
	*Oxybelis aeneus* (Wagler in Spix, 1824)	DS (Barreto et al. 2011)
	*Oxybelis fulgidus* (Daudin, 1803)	EO
	*Phrynonax poecilonotus* (Peters, 1867)*	EO
	*Rhinobothryum lentiginosum* (Scopoli, 1785) *	EO
	*Spilotes pullatus* (Linnaeus, 1758)	EO
	*Tantilla melanocephala* (Linnaeus, 1758)	Pitfalls
Dipsadidae	*Atractus alphonsehogei* Cunha & Nascimento, 1983	DS (Cunha and Nascimento 1983)
	*Atractus schach* (Boie, 1827)	DS (Cunha and Nascimento 1993)
	*Atractus snethlageae* Cunha & Nascimento, 1983	DS (Cunha and Nascimento 1993)
	*Dipsas catesbyi* (Sentzen, 1796)	EO
	*Dipsas variegata* (Duméril, Bibron & Duméril, 1854)	DS (Cunha and Nascimento 1993)
	*Erythrolamprus aesculapii* (Linnaeus, 1766)	DS (Cunha and Nascimento 1993)
	*Erythrolamprus cobella* (Linnaeus, 1758)	DS (Cunha and Nascimento 1993)
	*Erythrolamprus miliaris chrysostomus* (Griffin, 1916)	DS (Cunha and Nascimento 1993)
	*Erythrolamprus oligolepis* (Boulenger, 1905)	DS (Cunha and Nascimento 1993)
	*Erythrolamprus poecilogyrus* (Wied, 1825)	EO
	*Sibon nebulatus* (Linnaeus, 1758)	DS (Cunha and Nascimento 1993)
	*Sibynomorphus mikanii septentrionalis* Cunha, Nascimento & Hoge, 1980 **	EO (Freitas et al. 2014)
	*Imantodes cenchoa* (Linnaeus, 1758)	DS (Cunha and Nascimento 1993)
	*Leptodeira annulata* (Linnaeus, 1758)	EO/BA
	*Helicops angulatus* (Linnaeus, 1758)	DS (Barreto et al. 2011)
	*Hydrops martii* (Wagler in Spix, 1824)	DS (Cunha and Nascimento 1993)
	*Pseudoeryx plicatilis* (Linnaeus, 1758)	DS (Cunha and Nascimento 1993)
	*Philodryas olfersii* (Liechtenstein, 1823)	EO
	*Philodryas viridissima* (Linnaeus, 1758)	EO
	*Clelia plumbea* (Wied, 1820)	EO
	*Oxyrhopus melanogenys* Cunha & Nascimento, 1983	BA
	*Oxyrhopus petolarius* (Reuss, 1834)	EO
	*Pseudoboa coronata* Schneider, 1801	DS (Cunha and Nascimento 1993)
	*Pseudoboa neuwiedii* (Duméril, Bibron & Duméril, 1854)	EO
	*Pseudoboa nigra* (Duméril, Bibron & Duméril, 1854)	EO
	*Siphlophis cervinus* (Laurenti, 1768)	DS (Cunha and Nascimento 1993)
	*Taeniophallus quadriocellatus* Santos-Jr, Di-Bernardo & Lema, 2008	DS (Cunha and Nascimento 1993)
Dipsadidae	*Xenodon merremii* (Wagler in Spix, 1824)	EO
	*Xenodon severus* (Linnaeus, 1758)	DS (Cunha and Nascimento 1993)
Elapidae	*Micrurus filiformis* (Günther, 1859)	DS (Cunha and Nascimento 1993)
	*Micrurus hemprichii* (Jan, 1858)	DS (Cunha and Nascimento 1993)
	*Micrurus lemniscatus lemniscatus* (Linnaeus, 1758)	DS (Cunha and Nascimento 1993)
	*Micrurus spixii martiusi* Schmidt, 1953 **	BA
	*Micrurus surinamensis* (Cuvier, 1817)	DS (Cunha and Nascimento 1993)
Viperidae	*Bothrops atrox* (Linnaeus, 1758)	BA/EO
	*Bothrops brazili* Hoge, 1954	DS (Cunha and Nascimento 1993)
	*Bothrops taeniatus* Wagler in Spix, 1824	DS (Cunha and Nascimento 1993)
	*Lachesis muta* (Linnaeus, 1766)	DS (Cunha and Nascimento 1993)

## Appendix 1

**Specimens examined**

*Rhinella marina* CHP-UFRPE 1810 – *Rhinella gildae* CHP-UFRPE 1848 *Leptodactylus paraensis* CHP-UFRPE 1811-1845 – *Leptodactylus petersii* MZUSP – 154062 – *Lithodytes lineatus* CHP-UFRPE 1815 – *Leptodactylus mystaceus* CHP-UFRPE 1838-44-1863-64-1878 – *Physalaemus cuvieri* CHP-UFRPE 1834-37 – *Pristimantes fenestratus* CHP-UFRPE 1862-2042, MZUSP 154061 – *Phyllomedusa vaillantii* CHP-UFRPE 1874-2621 – *Hypsiboas punctatus* CHP-UFRPE 1877 – *Ctenophryne geayi* CHP-UFRPE 1820-1859-1876 – *Scinax ruber* CHP-UFRPE 1816 – *Dentropsophus marmoratus* CHP-UFRPE 1818 – *Trachycephalus tyfonius* CHP-UFRPE 1824-25 – *Adenomera andreae* MZUSP 154063-69 *Cercosaura argulus* CHP-UFRPE 2622 – *Amphisbaena amazonica* CHP-UFRPE 2065 – *Plica umbra* CHP-UFRPE 1826-28-1873 – *Iguana iguana* CHP-UFRPE 1870 – *Micrablepharus maximiliani* CHP-UFRPE 1866-69 – *Norops fuscoauratus* CHP-UFRPE 1817-1865 – *Tropidurus hispidus* CHP-UFRPE 1846 – *Copeoglosum nigropunctatum* CHP-UFRPE 1832-33 – *Gonatodes humeralis* CHP-UFRPE 1821-23 – *Coleodactylus septentrionalis* CHP-UFRPE 1819 – *Uranoscodon superciliosus* CHP-UFRPE 1813 – *Polychrus marmoratus* CHP-UFRPE 1856 – *Kentropys calcarata* CHP-UFRPE 1830-31-MZUSP 104280 – *Leposoma percarinatum* MZUSP 104278 – *Stenocercus dumerilii* MZUSP 104279 – *Cnemidophorus cryptus* MZUSP 104281 – *Salvator merianae* MZUSP-MTR 23332 – *Platemys platicephala* CHP-UFRPE 1814 – *Kinosternum scorpioides* CHP-UFRPE 2126 – *Amerotyphlops brongersmianus* CHP-UFRPE 1850 - *Anilius scytale* CHP-UFRPE 1860 - *Boa constrictor* CHP-UFRPE 2439 – *Corallus hortulanus* CHP-UFRPE 2457 – *Spilotes pullatus* CHP-UFRPE 1873 – *Leptophis ahaetulla* CHP-UFRPE 2267 – *Tantila melanocephala* CHP-UFRPE 1829 – *Sibynomorphus mikanii septentrionalis* CHP-UFRPE 1858-2234-38 – *Oxyrhopus pelotarius* CHP-UFRPE 2220 – *Oxyrhopus melanogenys* CHP-UFRPE 1829 – *Pseudoboa nigra* CHP-UFRPE 1852-53 – *Pseudoboa newiedii* CHP-UFRPE 1860 – *Erythrolamprus poecilogyrus* CHP-UFRPE 1875-1851 – *Leptodeira annulata* CHP-UFRPE 1847 – *Philodryas olfersii* CHP-UFRPE 1854 – *Dipsas catesbyi* CHP-UFRPE 1872 – *Micrurus spixii martiusi* CHP-UFRPE 1812 – *Bothrops atrox* CHP-UFRPE 1855.
